# A Dynamic Evaluation of the Process of Solving Mathematical Problems, according to N.F. Talyzina’s Method

**DOI:** 10.11621/pir.2023.0307

**Published:** 2023-09-30

**Authors:** Yolanda Rosas-Rivera, Yulia Solovieva

**Affiliations:** a National Pedagogic University, Mexico City, Mexico; b Autonomous University of Puebla, Mexico; c Autonomous University of Tlaxcala, Mexico; d Lomonosov Moscow State University, Russia

**Keywords:** math learning, mathematical concepts, mathematical thinking, math assessment, mathematical operations

## Abstract

**Background:**

The process of teaching mathematics represents a challenge for primary education, due to the different perspectives and disciplines involved. In addition, as an active and flexible process, it requires feedback on what the students actually achieved. An analysis of the different learning and development outcomes allows the teacher to understand the mathematical content and the method of teaching it in the classroom, with the objective of promoting the students’ conceptual development.

**Objective:**

The objective of our study was to analyze the general skills for problem solving which students developed, by applying dynamic evaluation.

**Design:**

A verification method was used to identify the students’ abilities and difficulties. A protocol for evaluating the process of solving mathematical problems was organized. The assessment included four simple problems and four complex ones. The participants were 15 students in the third grade of primary school attending a private school located in Mexico City.

**Results:**

The results showed that the students identified the types of mathematical operations (addition, subtraction, multiplication, and division) required to solve the problems as their objective. Therefore, their preparation of a solution plan, its execution, and its verification were based only on empirical thinking and quantitative information.

**Conclusions:**

We concluded that problem-solving is an intellectual activity that requires conceptual development to carry out a solution plan, execute it, and verify it, in addition to the main objective of answering the question posed by the problem. We propose that these characteristics be included in the organization of mathematics teaching in order to develop mathematical thinking.

## Introduction

The teaching and learning of mathematics together comprise a process of knowledge acquisition in which both teachers and students participate. Likewise, it requires various activities and actions with objects that facilitate such acquisition. There are several factors related to the teaching-learning process that have been investigated: for example, teaching practices (Weiss et.al., 2019); learning strategies (Reséndiz, Block, & Carrillo, 2013); the use of didactic materials (De Castro & Palop, 2019); the participation of non-formal knowledge; etc. Therefore, the problem-solving process involves a level of complexity that implies that teachers and researchers understand both mathematical content and the way it should be taught in the classroom, especially if it is in the early years, when a child’s thinking tends to be limited to the immediate characteristics of the reality around them (direct perception) (Vygotsky, 1993; 1995).

According to Cantoral (2006), the process of teaching and learning must include an object for interaction between the teacher and the students; the particular interaction proposed by these authors is the game. However, it is possible to identify those new important changes in teaching proposals, didactic materials, the use of technology, and the mathematical content to be taught (from algorithms to mathematical concepts), which are necessary to improve the whole process (Ávila, 2006). The results obtained from these modifications have not yet been evident in studies of teachers’ understanding of the process (Arévalo, 2015), the students’ learning (Rosas & Solovieva, 2019), or students’ psychological development (Solovieva et al., 2020; 2021).

In teaching mathematics, the teacher must not simply present the mathematical tasks, but also demonstrate the actions that the student must carry out in order to solve the tasks and obtain generalized learning (Talizina, 2019; Weiss, et.al., 2019). For this, the teacher must know the type of intellectual actions and mathematical concepts that are included in the mathematical tasks, specifically in the solution of mathematical problems. Intellectual action is understood as an action oriented towards a conscious objective, which cannot be reached by applying a direct and impulsive solution, or available in the domain of generalized relations and action procedures (Davidov, 1988). That is, according to the cited authors, the teaching of mathematics requires knowledge of the concepts of mathematics and intellectual development (Reséndiz, Block, & Carrillo, 2013; Rosas & Solovieva, 2019; Talizina, 2019; Weiss, et. al., 2019).

The functional parts of intellectual actions identified in the studies by Talyzina (2017; 2019) are as follows: orientation, execution, and verification. There are investigations that have proposed the ways to formulate and develop intellectual actions through solving problems (García & Tintorer, 2016; Granados & Rodríguez, 2011; Nikola & Talizina, 2017; Rosas, Solovieva, & Quintanar, 2017; Volodarskaya, 2017). García and Tintóreo (2016), Granados and Rodríguez (2011), Nikola and Talizina (2017), Rosas, Solovieva and Quintanar (2017) and Volodarskaya (2017) have implemented teaching methods for formulating the process of problem-solving in basic education. Their proposals include a mathematical content (arithmetic, algebra, or geometry) and the identification of mathematical concepts in different contextual situations. The use of these methods allows us to identify all the students’ procedures which lead to a solution.

## The process of solving mathematical problems

According to activity theory as applied to teaching, it is possible to understand and study thinking as an inherited formal function that is used to solve mathematical problems, or the content of a system of intellectual actions that are developed during the solution of mathematical problems, and evolved through a series of stages (Nikola & Talizina, 2017). Nikola y Talizina (2017) believe that general thinking skills can be assimilated in one of two ways: the spontaneous method and the directed one. The spontaneous method consists in applying habits of thought not conceived as specific objects of assimilation; these habits are the means of the action. They do not have a reflective or conscious character; in addition, their acquisition occurs from the assimilation of knowledge and solving specific problems, so the results cannot be generalized. Thus, they are limited in their application.

The directed method requires consideration of the thinking skills involved as objects of assimilation. The process of forming thinking skills is significantly reduced in time because it has a directed and organized nature. It is possible to identify both pathways of assimilation in the forms and methods of teaching mathematics in classrooms involved in basic education (Reséndiz, Block, & Carrillo, 2013; Rosas & Solovieva, 2019; Weiss et al., 2019). Therefore, it is necessary to reflect on our theoretical perspective.

Mathematical problems require the knowledge of applied mathematics itself and involve situations that include a topic expressed in the language of mathematics. Also, it is possible to identify the basic elements of mathematical problems and understand their relationships. Some topics addressed in mathematical problems involve situations that relate to students’ everyday experiences; for example, buying and selling. Students can solve problems of this type without understanding the specific mathematical meaning of the concepts of “price, value, quantity, and product.”

However, there are other topics that are studied in schools, which are difficult to relate mathematically to everyday experience, as is the case with topics like “movement,” “velocity,” “volume,” and “distances,” or theoretical situations that involve the proof and application of theorems, as in the case of geometry (Butkin, 2017). Therefore, it is necessary for the teacher to organize the essential actions and direct the student’s attention to them so that they can understand and successfully solve any mathematical problem, regardless of the theme and their daily experience.

According to a psychological analysis of the problem-solving process, the activity of solving mathematical problems has a psychological structure. It is necessary to consider this structure in order to organize the complete orientation and direct the students. The structure goes as follows: 1) an objective, which consists of answering the question posed; 2) understanding the conditions; 3) retention of information; 4) elaboration of a general plan or solution strategy; 5) execution of the plan; and 6) verification of the solution (Luria & Tsvetkova, 1987; Tsvetkova, 1999).

It is also necessary to mention that mathematical problems have various characteristics: 1) they can be simple or complex, depending on the operations that are necessary (direct or intermediate); 2) they can establish known or new mathematical relationships (I have solved a similar problem or it is the first time it is presented to me); 3) they can be problems designed by the teacher, by a classmate, or by oneself; 4) they can be presented in writing, audio-verbally, or dictated; or 5) they can have a solution or not have a solution. Problems without a solution are those which don’t include complete information, have insufficient information provided, include information unrelated to the final question, or do not pose a question at all (Luria & Tsvetkova, 1987; Tsvetkova, 1999).

In summary, the study of problem-solving requires both specific knowledge of mathematics and of the psychological actions involved in this process. Therefore, the objective of our study was to analyze the thinking skills developed by students to solve mathematical problems in a typical primary school in Mexico. A verification method was used to identify the abilities and difficulties of the students through a dynamic evaluation based on the application of activity theory to teaching (Talizina, 2000; 2017; 2019). The results are intended to contribute to reflections on the teaching of mathematics and its objectives.

## Methods

The method used in the study was experimental verification as proposed by Talyzina (2000; 2019). According to Talizina (2000), the experimental method is characterized by: 1) the establishment of an objective; 2) planning the steps of the experiment; 3) carrying out the experiment; 4) analysis of the data obtained by the researchers; and 5) the conclusions that the data allow the researchers to reach.

The verification method aims to characterize the current state of existing phenomena. It also allows the identification of the starting level of knowledge and skills for the assimilation of a concept (Talizina, 2000, 2017; Nikola & Talizina, 2017).

### Participants

The selection of the participants was intentional. The private school selected for the study is located in Mexico City. The school uses the programs of the Secretary of Public Education as the main teaching method. This school was also considered because it has only one group of third graders and only 22 students in total. Such a number of pupils facilitates participation in research projects that aim to analyze and improve the process of teaching and learning of mathematics. The main pedagogical approach, used by the Secretary of Public Education in Mexico, is that of competences, which are supposed to be acquired through key learning (SEP, 2017). The third grade of primary school was selected because the Basic Education program establishes that it is in this grade that the four basic mathematical operations must be learned, and problem-solving is proposed for teaching them.

A private school was chosen because it was possible to get an agreement to allow educational research aimed at improving its teaching methods. The school is located in the southern district of Mexico City. The participants were 15 regular students of the third grade of primary school: five boys and ten girls. The students had an average age of 8.5 years. Nine students (five girls and four boys) got high marks in mathematics (a 10 on a scale of 1 to 10). Six students (five girls and one boy) got a mark of 7 (same scale), and the teacher reported them having some difficulties in learning mathematics (solving of mathematical operations, mental calculations, and understanding mathematical problems).

### Procedure

The research was organized in the following phases: 1) selection of the participants — the research project was presented to the school director to organize the application dates; 2) design of assessment (protocol and materials) — selection of the tasks which were based on previous formative studies (Rosas, 2013; Rosas, Solovieva & Quintanar, 2019); and 3) individual evaluations.

The evaluation phase was carried out individually in the classroom, with no external distractions. Each evaluation lasted approximately one hour. The evaluator went to the classroom and asked the group teacher for authorization to work with each student. Then, the evaluator asked the student to sit down and welcomed him or her. Subsequently, there was a friendly interaction to find out the student’s name and some of his or her interests in mathematics. Also, a general idea of the study was explained to him or her.

The content of the evaluation consisted of reading a problem at least three times to each student, and asking him or her to write down what was needed to solve the problem. Finally, the student had to explain the method for finding the correct answer to each problem and the whole process of solution. If the students had doubts, several kinds of support were provided: verbal (mathematical explanation, explanation of the structure of the problem, repetition of information or reflective questions about the content of the problem); perceptual (concrete drawing of the conditions or elaboration of diagrams); or materialized (use of sticks for arithmetical operations).

### Instrument of evaluation

The evaluation was organized based on the works of Nikola & Talyzina (2017), Talyzina (2017), and Tsvetkova (1999). The protocol proposal has been previously published in works by Rosas, Solovieva, and Quintanar (2019). The present publication presents the tasks that correspond to the topic of solving mathematical problems.

The tasks of the protocol consisted of: a) simple problems (may require an operation to be solved) and complex problems (may require more than one operation to be solved); b) problems requiring the four basic mathematical operations (addition, subtraction, multiplication, and division); c) problems featuring thematic situations — processes (distance), buying and selling, distribution, increase and decrease of a measure; d) problems with and without solutions; and e) problems with both quantitative and qualitative questions. According to Talyzina (2017) and Tsvetkova (1999), considering this range of characteristics makes it possible to identify general thinking skills.

The following are the problems we presented to the children.

*Simple problems:*

- The train has covered the distance of 98 km in 11 hours; how many km does the train travel in one hour? (Division)
- The library “The little prince” contains 40 books, which are distributed on 5 shelves. If teacher Lupita puts the same number of books on each shelf, how many books are on each shelf? (Division)
- There were 19 chocolates in the box. The children ate some of the chocolates and 11 were left; how many chocolates did they eat? (Subtraction)
- For 12 days, 48 km of the road were built; how many cars passed during a day? (no solution).

*Complex problems:*

- Renata and Daniel went to the market and bought the following items: 2 kilograms of apple, 300 grams of sugar, and 1 kilogram of pasta; how many grams did they buy in total? (conversion of measurements/ addition)
- Gaby is three times as old as her sister Sofía. If Sofía is 7 years old, how old is Gaby? (multiplication/ more times)
- In the Children’s Museum, the first room has 64 play activities, and the second room has four times less. How many play activities are there in the second room? (division/ less times)
- Axel and Daniel played three rounds of penalties. If in the first round Axel scored 20 respectively goals and Daniel scored 18 goals, in the second round they scored 35 to 20 and in the third 15 to 50, who won? With how many goals? (qualitative-quantitative question).

## Results

First, the students expressed that their liking for mathematics and the way their teacher teaches it. One student commented that her teacher was nice and that she liked to go to the blackboard to solve problems. Another student mentioned that her teacher explains a problem to her several times when she does not understand it. The children did not show any concern in relation to the difficulties they had during their work with the experimental protocol; they expressed interest in solving the problems in all the proposed tasks. However, they did not ask questions, they did not request support, and they waited to be told what they should do to resolve their difficulties.

The preliminary results were described by the number of problems solved correctly, incompletely, and incorrectly. Subsequently, the types of errors made by the students were described and organized according to the types of problems they had.

In the process of solving the simple problems, all the pupils were able to identify and to solve the mathematical operation of the subtraction problem (decrease) and the division problem (distribution), but they were unable to answer the question posed by the problem. In addition, no student managed to solve the problems of processes (distance), and the one without a solution.

In solving the complex problems, all the pupils had difficulties in identifying the intermediate operations (measurement conversion, less times-division and more times-multiplication). Although the students were able to carry out the intermediate operations with the help of an adult, they proceeded without understanding and never reached the solution.

These general results allowed us to identify the students’ specific difficulties during the process of trying to solve the problems. These difficulties were: 1) difficulties in actually answering the question posed, instead of which the students only identified the mathematical operations needed for a solution; 2) difficulties in understanding the intermediate and non-immediate operations; 3) difficulties in identifying the mathematical operations and the solution of the algorithm; and 4) difficulties in counting.

The results are presented below for each type of the problem, along with a description of the types of errors and the types of help which were provided to the students during the process of solving the problems.

In the simple problems (*Table 1*), in general, the students had difficulties in counting, using and converting the decimal number system, identifying the measuring unit, and completing the answer to the problem. The students were able to solve the problem about the library (division) and the chocolates (subtraction) incompletely. The students counted verbally and with the help of their fingers, although they did not answer the final question the problem posed; they only identified the result of the mathematical operation. In addition, the students made mistakes in the two problems involving data on distance and time processes.

**Table 1:** Answers to the simple problems

Type of problems	Answers	Types of errors	Types of support
A train traveled 98km in 11 hours; how many km does the train travel in one hour?	Impossibility 48 11	Difficulty understanding the relationship between data Difficulty organizing the data in the algorithm Difficulty understanding the processes of distance and time	Mathematical explanation of the data through the schematic drawing Organization of the data in the algorithm, writing of the decimal numerical system
A 48-km road is being built; how many cars pass during a day?	None because they were building it 12 + 48 1 4 48	Difficulty in analyzing and relating the parts of the problem Difficulty finding other ways to solve	Explanation and division of the structure of the problem Reflexive questions towards the structure of the problem

*Note. Table 1 contains the answers of the pupils to the simple problems.*

In the problem about the train, the students had difficulties imagining the situation of the problem; they focused on the content of the train, on describing what it was like, and expressing their interest in it. For example, some students said they had seen a train but not had the experience of traveling on one. The type of help consisted of reflecting on the situation and understanding its quantitative relationship; that is, the train was not important but what was happening with the train. Once they understood what was happening, they were able to identify by a process of elimination that the strategy needed was division. For example, the students said addition was not useful, because the problem does not ask for a total of something; nor was subtraction useful. The children used division: it would be 98 divided by 2, and thus they obtained the result of 48 (miscount). Later they commented that the answer was 11 because the data on the hours was included.

The second type of help given to them was a kind of representative perceptual support, based on drawing two points to represent the start and end of the distance covered by the train, and the line to represent the path of the train. The students understood the data that they had to find, but they did not know how to organize the data in the division algorithm; so they were oriented to describing the components of the division and placing the corresponding data. Finally, the students made mistakes in counting. The children solved the multiplication problems verbally, but they had counting errors so they were helped to verify their counting by writing the partial results.

The second common difficulty was the problem which had no solution (the last simple problem). The students did not identify the elements of the problem, but focused only on the numbers. They proposed to answer with addition or with the repetition of the quantitative data of the problem, thinking that the solution was embedded in the problem. The verbal support of an adult presented the structure of the mathematical problem: the description of a situation and the question posed by the problem. With such support, the students were able to understand that each part of the problem consisted of specific information, which must be related to the final question. With the help of reflective questions, students had to understand what situation posed the problem? Who is he talking about? What data is mentioned? How should the solution to the problem be planned? What is the question that must be answered? What do we want to know or find in the problem? Do we have enough information to answer this question?

*Table 2* presents the difficulties the students had while trying to solve the complex problems. They had significant difficulties understanding the mathematical operations required for intermediate actions; for example, those of conversion to the decimal number system, or identifying the operation of division as the reduction of a given measure to a quantity of time. In the problems with the conversion of the weight measurements, the students operated directly with the data. Although when they were asked about the relationship of a kilo to grams, the students could say that a kilo is equal to a thousand grams, in the context of the problem, they could not identify how the corresponding conversions should be performed. So, the first type of support was to write the equivalence; the second support was to identify the measurement in which the data was presented and the corresponding conversion, which was required to answer the question posed by the problem. Subsequently, the data was organized correctly, and intermediate operations were added. Finally, the students answered the problem.

**Table 2:** Answers to complex problems

Type of problems	Answers	Types of errors	Types of support
Renata and Daniel went to the market and bought the following: 2 kilos of apple, 300 grams of sugar and 1 kilo of pasta. How many grams did they buy in total?	603 6000 301 303 600	Difficulties in identifying measurement and conversion Identify the hierarchical value of the number	Explanation and writing of the decimal number system Organization of the data in the algorithm; writing of the decimal numerical system
Gaby is three times as old as her sister, Sofía. If Sofía is 7 years old, how old is Gaby?	10 4 22 20	Difficulty understanding the relationship between data Difficulties in identifying the mathematical operation Counting difficulties	Reflexive questions towards the information of the problem Explanation and division of the structure of the problem Explanation of the multiplication operation
In the children’s museum, the first room has 64 play activities and in the second there are 4 times less. How many play activities are there in the second room?	160 60 200	Difficulty in analyzing and relating the parts of the problem Difficulty finding other ways to solve the problem	Explanation and division of the structure of the problem Reflexive questions towards the structure of the problem
Axel and Daniel played three rounds of penalties. If in the first round the score was 20 to 18 goals respectively, in the second 35 to 20, and in the third 15 to 50, who won? for how many goals?	Daniel for 10 goals Daniel because he made 50 more Second won, scored 8 more	Difficulty in organizing the data Responded impulsively	Write the data in order Reflexive questions towards the structure of the problem

*Note. Table 2 contains the answers of the pupils while trying to solve the complex problems.*

In the problem about Gaby’s age, the students had difficulty understanding the relationship between the data. Some students used the addition operation directly, other students responded with multiplication but could not explain why they used multiplication. There were also difficulties in counting and verifying their responses. The type of support that was given to them was a mathematical explanation of multiplication and an analysis of the situation of the problem so that they understood the reason for using the multiplication operation. However, no one answered the problem completely; they only mentioned the result of the multiplication.

In the problem about the children’s museum, a division operation is posed, which is expressed by the term “less times,” which does not imply a direct subtraction but the use of division. Some students responded with direct subtraction (64–4 = 60); others tried an operation of multiplication (4 times 40, or 4 times 50). The type of support that was given to them consisted in an analysis of the relationship between the data, along with the explanation that the decrease should not be performed directly but with the help of the given measurement. The students understood the importance of using division. Although they had difficulties solving the problem with the division algorithm, the students were confused about the decimal number system of the result and in the location of the quotient and the remainder.

In the problem about Axel and Daniel, the students had difficulty organizing the information. They combined the quantitative data, so they had to write the data in two columns. They also focused on the final data (50 goals) to answer who was the winner; however, they could not identify the difference between the goals. Therefore, the students had to receive support to identify the information, organize it, and respond through mathematical operations.

Finally, the results obtained in the study showed that the students have developed the following thinking skills: 1) identification of an objective: the mathematical operation; 2) elaboration of the plan: identification of the direct mathematical operations needed (addition, subtraction); 3) execution of the plan: choosing mathematical operations with an estimation strategy, despite difficulties in the conversion of measuring units and in understanding the decimal numerical system, and incomplete solution of algorithms; and 4) verification of the result. (But they only verified the quantitative operations, without actively reflecting on their strategies.

## Discussion

The objective of this research was to analyze the thinking skills developed by a group of primary school students (private school, third grade). Based on the proposed dynamic evaluation, the following characteristics of their thinking skills were identified, which might be described as: 1) the empirical nature of the process of solution; 2) the use of habits and mechanized actions for solving problems; 3) the reproduction of actions with no reflection; and 4) problem solving without some kind of intellectual activity by the pupils. These characteristics of thinking are related to the general social interaction which the students engage in both at school and in their daily activities. According to Davidov (1988) and Talyzina (2017), this shows that the school is not developing the theoretical thinking that mathematics knowledge requires.

The data we obtained allows us to observe that the students acquired their knowledge from the immediate experience of reality, both in class and in their daily lives, and that the operations they used were quantitative; this information coincides with the study carried out by Reséndiz, et al. (2017). For example, one student who helped sell products in his father’s establishment, had acquired the habit of recognizing the results of multiplication operations (for example, 7 times 3, 40 times 5). Another student mentioned that her teacher had taught her strategies for solving problems, which consisted of interpreting key words (*i.e.,* total = addition, by = multiplication, and between = division). However, both students had difficulties when carrying out operations which used the formal multiplication and division algorithm. When the algorithms of the four mathematical operations are taught in elementary school, each algorithm has a sequence of operations to achieve a result. In the case of division, it is possible to use the subtraction algorithm or the multiplication algorithm.

According to Salmina (2017), students can solve mathematical operations mechanically or by using memory because they may use some operations that might have been used in their daily lives. At the same time, the same pupils might have symbolic and logical difficulties on problems that require operating according to mathematical concepts and signs. In addition, in the evaluations, the students made mistakes in understanding and handling the decimal number system. The pupils had difficulties with the use of materialized supports (use of sticks for proper counting) and in the conversion of measuring units. The most effective type of support was the use of drawings to represent the concrete situations, mentioned in the problems.

The second characteristic was the use of habits and mechanized actions without reflection on the logic of the problems during the process of finding the solution. The students could not reflect on their actions; they did not understand what information was relevant and what was irrelevant to the problems. The students only proposed to find the correct mathematical operation and to solve the problem directly, even if they did not understand what the whole problem was about and the quantitative relationships between the data.

In the problem with the train, for example, the students selected the operation of division because they tried to use direct operations of addition, subtraction, and multiplication unsuccessfully. They did not understand the quantitative relationship, which depended on the use of the measurements according to a specific ratio (km/ hr). The children were only focused on the object (the train) and the quantities (the numbers mentioned in the problem). Despite provision of perceptual support (drawing a schematic for the problem), the pupils could not identify which part of the drawing represented the distance and which represented the period of time. Also, the students verified their answers with the results of the mathematical operations. For example, they commented that “the result is 8 because 98 divided by 11 is 8,” and they did not use decimal numbers or the remainder (the sign) in the operation of division. They never noticed their misunderstanding of the problem and the mistakes related to this misunderstanding.

According to the results, the pupils didn’t understand the relationship between physical magnitudes, and even the external help of an adult didn’t allow them to arrive at the solution to this problem. The prior understanding of physical concepts, especially that of process and its duration, is necessary for solving math problems in primary school (Obukhova, 1968), but the traditional educational program doesn’t include these processes, which is one of the great obstacles to the children understanding the process of problem-solving at school.

In light of the analysis by Cantoral et al. (2005) on the process of problem-solving, it is possible to observe the chilren’s predominant use of daily isolated situations instead of generation of mathematical and conceptual thinking. Such daily isolated situations are preferentially used by practically all teachers as a strategy of working with the problems. The teachers in the classroom do not spend time to generate argumentation, create strategies, or provoke self-reflection. This makes it difficult for the student to ask himself: what should I do? How do I think about the problem? Do I have another way to solve it? How do I know that I achieved the right result? What mistakes did I make? How did I correct them? and so on. In addition, Arévalo (2015) mentions that the prevalence of individual work hinders students from being able to ask questions and be able to support themselves for learning.

The third characteristic is the tendency to repeat one’s previous actions. The students frequently relied on a strategy of trial and error, which means that they had problems in counting and in understanding the mathematical operations. Their main route was to repeat operations for each problem with no reflection on their use. The pupils knew the steps that must be carried out in the algorithms. With no clear understanding of the structure of the decimal number system, they made spatial errors (confusion about the location of the digit in the structure of the number, confusion of the minuend and subtrahend, confusion about the conversion of measurements). These errors were corrected when the elements of the structure of number were explained to the students. This structure might be represented as the magnitude, the unit of measurement, and the number of times the measurement was used (Salmina, 2017; Talizina, 2017; Rosas, 2019).

One type of help consisted of writing each number in the decimal system. With this help, the students understood that the operations which they called “borrowing” are conversions of the measuring units. The formation of theoretical mathematical concepts allows the student to understand the various connections and actions between the elements in the structure of number. In this case the concept of number allows the students to understand the mathematical actions and to create their own mathematical problems (Rosas, 2019).

Finally, the pupils’ problem-solving did not represent any kind of intellectual activity. Their main objective was to find the mathematical operation and to solve it. Thus, their actions were impulsive, their results were incomplete, and they had difficulty in solving complex problems. According to Luria and Tsvetkova (1987), Nikola and Talyzina (2017), Tsvetkova (1999), and Solovieva (2022), the process of problem-solving aims to answer the question posed. The question will guide the student to select whatever information is relevant to the problem and whatever is not. For example, in the problem with no solution that was used for the evaluation, the students had difficulties understanding that a problem can have no quantitative solution at all. In addition, the pupils carried out several mathematical operations in an effort to solve this problem. If they had attended to the question, then they would have avoided such failed efforts.

This type of error indicates the absence of consistent work with the students to organize their intellectual activity. This absence is common in primary schools and shows the deficiencies in teaching method not only for problem solving, but also for the introduction of mathematical concepts. The relevance of developing thinking skills in primary school is an objective of psychological development that education must achieve; this has been proposed by Vygotsky (1995) and his followers (Davidov, 1988; Galperin, 2009; Talizina, 2017). In addition, in other countries, such as Colombia and Brazil, this line of studies was continued (García & Tintóreo 2016; Granados & Hernandez, 2011; Rosas, 2013; Rosas & Solovieva, 2019).

The authors of this article also consider the students’ development of scientific concepts to be a predictor of overall successful learning in basic education. Although the students managed to mechanize some actions, their difficulties showed up in their lack of understanding of situations that are not related to direct perception. In our study the problem that the children had the greatest difficulty understanding was the train route, because the students had seen a train but could not represent the time and the relationship with the distance covered.

In summary, the results of our study allowed us to discover that the students participating in the evaluation showed empirical thinking, produced by habits, practical experience, and the tendency to repeat their previous actions. These results lead us to propose work with mathematical concepts in primary education and the organization of the process of problem-solving as an intellectual activity. The introduction and gradual development of intellectual activity may have a positive effect on the students’ psychological development. Conceptual thinking represents a new qualitative stage in a child’s development (Vygotsky, 1993, 1995). In addition, incorporation of various types of external help may encourage the students to access a reflective solution. External help might be presented at different levels of the actions: material, perceptual, and verbal (Solovieva, 2022).

In Mexico, students are evaluated only quantitatively (Arévalo, 2015; Rosas, 2019; Weiss, et al., 2017). It is thought that learning success is related to mental actions and taking less time to reach a solution (quick solutions). The teaching of mathematics is seen as the repetition of operations in an abstract way with no relationship to the situations presented by the problems, which are considered as practical solutions of day-to-day life. In other studies (Rosas, 2013; Rosas & Solovieva, 2019), the lack of a conceptual explanation of mathematical content was observed during the process of teaching in primary schools. The digits were only associated with the counting of concrete objects. For example, the use of one cube assumes the value of 1 unit, while the cubes of another color represent the unit of 10 or 100. When the colors were changed or absent, the pupils became unable to carry out any kind of operation (Rosas & Solovieva, 2019).

According to Talyzina (2017; 2019), traditional teaching calls for the formation of absolute concepts and abstract answers. For example, it is thought that if a student achieves mental calculations in less time, he or she is capable in mathematics. In our study, the insistence of the students on solving the tasks mentally and quickly with no reflection was also observed. According to Talyzina (2017) and Galperin (2009), before the work on mental level, problem solving must be formed on different levels of execution: material, perceptive, and external verbal. Traditional education never used these levels of intellectual actions, so that the pupils may only memorize direct mechanical solutions.

The data from our study shows that another means of teaching and evaluation of learning exists. Dynamic evaluation shows the possibility and necessity of using intellectual actions on different levels, such as level of materialized, perceptive, and verbal actions (Veraksa, et al., 2022). The important aspect of solving a problem is the understanding of the conceptual content of the problem, so that the student may reflectively act with each element of the problem, not in isolation but jointly as a complex intellectual action (Solovieva, et al., 2021; Solovieva, Quintanar, & Sidneva, 2023).

## Conclusion

The first conclusion relates to the use of dynamic evaluation. Dynamic evaluation allows us to provide types of external help by an adult. In addition, the possibility of constant interaction of the teachers with the students tends to increase the students’ level of motivation. They become aware of their actions and their mistakes. The various types of external help allow them to understand what they are doing. So, dynamic evaluation impacts cognitive and affective development, in this case, cognitive interest in mathematical knowledge.

The second conclusion concerns the content of the problem-solving process in primary school. In this study, the problem-solving used in primary school can be viewed as a mathematical task, not as an intellectual activity of the pupil. Therefore, the students participating in the study were only able solve problems as a means of practicing mathematical operations, not as a way of developing concepts and applying them in different situations (everyday, theoretical, empirical, etc.)

Lastly, activity theory applied to teaching provides methodological theoretical tools for the study of the learning-teaching process of mathematics. The works of Talyzina (2017; 2019) and Nikola & Talyzina (2017) provide both a conceptual knowledge of mathematics, and of psychological development and pedagogical forms of work in the classroom. The results of our study show the possibility of providing necessary external help not only during the process of solving problems, but also earlier, starting from the formation of the concept of number and the decimal system (Rosas, 2019; Rosas & Solovieva, 2019; Veraksa et al, 2022).

## Limitations

The limitation in this investigation is related to the possibility of generalizing the results from such a small sample. Work with larger populations would allow us to characterize the process of mathematical problem-solving more completely and provide more complex strategies.
